# Growth Differentiation Factor-15 as a Biomarker for Sarcopenia in Patients With Chronic Obstructive Pulmonary Disease

**DOI:** 10.3389/fnut.2022.897097

**Published:** 2022-06-30

**Authors:** Mingming Deng, Yiding Bian, Qin Zhang, Xiaoming Zhou, Gang Hou

**Affiliations:** ^1^Department of Pulmonary and Critical Care Medicine, Center of Respiratory Medicine, China-Japan Friendship Hospital, Beijing, China; ^2^Graduate School of Peking Union Medical College, Peking Union Medical College, Chinese Academy of Medical Sciences, Beijing, China; ^3^National Center for Respiratory Medicine, Beijing, China; ^4^Institute of Respiratory Medicine, Chinese Academy of Medical Sciences, Beijing, China; ^5^National Clinical Research Center for Respiratory Diseases, Beijing, China; ^6^Department of Pulmonary and Critical Care Medicine, First Hospital of China Medical University, Shenyang, China; ^7^Respiratory Department, Center for Pulmonary Vascular Diseases, Fuwai Hospital, National Center for Cardiovascular Diseases, Chinese Academy of Medical Sciences, Peking Union Medical College, Beijing, China

**Keywords:** chronic obstructive pulmonary disease, sarcopenia, GDF15, biomarker, nomogram

## Abstract

**Purpose:**

Sarcopenia is an important factor contributing to comorbidities in patients with chronic obstructive pulmonary disease (COPD) and is an independent risk factor for increased mortality. The diagnostic process for sarcopenia requires specific equipment and specialized training and is difficult procedurally. A previous study found that GDF15 levels are associated with skeletal muscle mass and function in patients with COPD. However, whether circulating GDF15 levels can be used for the prediction of sarcopenia in patients with COPD is unknown.

**Methods:**

This study included 235 patients with stable COPD who were divided into a development set (*n* = 117) and a validation set (*n* = 118), and we followed the definition of sarcopenia as defined by the guidelines from the Asian Working Group for Sarcopenia. Serum concentrations of GDF15 were measured using an enzyme-linked immunosorbent assay (ELISA), and construction of a nomogram and decision curve analysis were performed using the R package “rms.”

**Results:**

In this study, serum GDF15 levels were negatively associated with skeletal muscle mass (*r* = –0.204, *p* = 0.031), handgrip strength (*r* = –0.274, *p* = 0.004), quadriceps strength (*r* = –0.269, *p* = 0.029), and the thickness (*r* = –0.338, *p* < 0.001) and area (*r* = –0.335, *p* < 0.001) of the rectus femoris muscle in patients with COPD. Furthermore, the serum levels of GDF15 in patients with sarcopenia were significantly higher than those in controls. Importantly, serum levels of GDF15 could effectively predict sarcopenia in patients with COPD based on the development set (AUC = 0.827) and validation set (AUC = 0.801). Finally, a nomogram model based on serum GDF15 levels and clinical features showed good predictive ability (AUC > 0.89) in the development and validation sets.

**Conclusion:**

Serum GDF15 levels could be used to accurately and easily evaluate sarcopenia in patients with COPD.

## Introduction

Chronic obstructive pulmonary disease (COPD) is a persistent, progressive, chronic airway disease (1) that represents a major global health burden, causing more than three million deaths worldwide, and represents the third leading cause of death (2, 3). COPD is primarily characterized by persistent airflow limitation and a variety of respiratory symptoms, and patients often develop systemic complications, such as cardiovascular disease, osteoporosis, anxiety and depression, as well as respiratory complications, such as lung cancer and asthma (4, 5). Sarcopenia is an important comorbidity in patients with COPD, with a prevalence of up to 15–55% (6, 7), and is mainly manifested as progressive and widespread loss of muscle function and is an independent risk factor for increased morbidity and mortality (8).

Early identification of sarcopenia can assist physicians in clinical management and improve prognosis, which are essential for the management of COPD patients. The diagnosis of sarcopenia requires simultaneous measurement of skeletal muscle mass (dual-energy X-ray or bioelectrical impedance analysis) and skeletal muscle function (grip strength and walking speed) (9, 10). The diagnostic process of sarcopenia requires specific equipment and specialized training and is relatively difficult to perform in developing countries and primary care settings. The SARC-F and calf circumference have been used to screen for sarcopenia (11, 12). Muscle function tests also show potential clinical value in screening sarcopenia, such as the 5-time chair stand test (5STS) for strength and walking tests for physical performance (13, 14). In addition, the measurement of circulating biomarkers is inexpensive and could be routinely performed in clinical practice and could be a good complement to the above methods.

Growth differentiation factor-15 (GDF15), which belongs to the large family of growth differentiation factors, is a member of the transforming growth factor β superfamily (15). GDF15 expression is usually significantly elevated in response to histopathological damage, such as ischemic diseases (16), cancer (17), and cardiovascular disease (18), making it a potential disease biomarker. Circulating GDF15 levels increase with age, where high levels are strongly associated with an increased risk of death (19). Several studies suggest that elevated levels of circulating GDF15 are strongly associated with poor muscle function and physical function in healthy individuals (20) or patients with chronic disease (21, 22) and may serve as a biomarker for older adults in the community, patients with myositis (23), or patients with cardiometabolic disease (24). GDF15 has been explored in only one study in relation to muscle mass in patients with COPD (25), and whether circulating GDF15 levels can be used to predict sarcopenia in patients with COPD is unknown.

The aim of this study was to assess the clinical value of circulating GDF15 in prospectively screening for sarcopenia in patients with COPD and to determine the cutoff value for use in clinical practice.

## Materials and Methods

### Study Design and Patients

This is a prospective multicenter cross-sectional study. Patients with stable COPD were recruited at the First Affiliated Hospital of China Medical University (Shenyang, China) and the First Affiliated Hospital of Dalian Medical University (Dalian, China) from August 2018 to December 2019.

The inclusion criteria were as follows: diagnosis of stable COPD according to the Global Initiative for Chronic Obstructive Lung Disease (GOLD) criteria; age ≥ 40 and < 80.

The exclusion criteria were as follows: (1) exacerbation of COPD within the last month; (2) combination of severe cardiovascular disease and active lung disease; (3) long-term application of systemic steroid therapy; and (4) inability to read or understand the informed consent. The study was approved by the research ethics committees of the First Hospital of China Medical University (No. 2018-144-2) and was approved by ethics committees at the First Hospital of Dalian Medical University.

A total of 117 patients with COPD who were recruited from the First Hospital of China Medical University were enrolled in the development set to assess the clinical value of GDF15 and determine the cutoff values. Another 118 patients with COPD who were recruited from the First Hospital of Dalian Medical University were enrolled in the validation set.

### Data Collection

In this study, all measurements were performed at one time point, and the general characteristics of the patients, such as age, sex, and smoking status, were recorded. Spirometry was performed according to the American Thoracic Society and European Respiratory Society guidelines using the Jaeger MasterScreen system (Jaeger, Viasys Healthcare GmbH, Hoechberg, Germany). Dyspnea symptoms and health status were measured using the modified British Medical Research Council (mMRC) dyspnea score (26) and COPD assessment test (CAT) (27), respectively.

### Five-Time Chair Stand Test

The 5STS was used to assess physical performance following a previous study (28). Specifically, patients were asked to perform in a 48 cm high armless chair with their feet on the ground, back pressed against the back of the chair and hands folded in front of their chest. After the researcher gave the command to start the test, the patient completed five standing and sitting movements as fast as possible, and the time was recorded. During the test, the patient’s arms had to be crossed over the chest, and the knees had to be fully extended while standing. During the test, the researcher verbally encouraged the patient. Patients performed three 5-STSs with the appropriate number of intervals. The 5STS test was conducted three times following our previous study (28) and other study (29). The minimum value of three trials of the 5STS was considered the participant’s score.

### Six-Minute Walking Distance

Exercise tolerance was determined using the 6-min walking distance (6MWD) following the 2002 American Thoracic Society (ATS) guidelines (30). A closed, long and straight 30-m corridor was chosen for the test. The test method was explained to the patient before the test, and the patient was told to walk as much as possible. If patients feel shortness of breath, chest pain or dizziness, test personnel should encourage them to slow down or stop and rest. If these symptoms worsen or persist even after rest, the test should be stopped immediately. After 6 min, the patient was stopped on command, and the distance (6MWD) traveled in meters was recorded.

### Quadriceps Muscle Strength

Quadriceps muscle strength in patients with COPD was measured using a dynamometer (microFET2™; Hoggan, Salt Lake City, UT, United States). The procedure followed the manufacturer’s instructions for use and our previous studies (31, 32). In short, the patient’s knee was flexed at 90°, and a dynamometer was placed. The front end of the dynamometer is located 5 cm proximal to the lateral malleolus on the anterior surface of the leg, perpendicular to the long axis of the tibia. The medical staff held the force on the force gauge plate and told the subject to push the plate with maximum knee extension and hold it in the same position for 4 s. The same procedure was repeated twice, 30–60 s apart. The average of the two assessments for each lower extremity was recorded as the maximum unilateral contraction force. The average of the bilateral contraction forces was then used as the quadriceps strength.

### Ultrasound Measurement of Rectus Femoris

Measurement of rectus femoris thickness and cross-sectional area followed our previous studies (31, 32). In short, grayscale ultrasound scanning was performed using the Aixplorer ultrasound scanning system and a 4–15-MHz linear array transducer (5 cm width). Patients rest quietly for 15 min before the test and then lie on their backs and relax all muscles. Our ultrasonographers are properly trained and have more than 10 years of experience. The ultrasound probe was placed perpendicular to the patient’s dominant leg. The transducer is positioned perpendicular to the long axis of the dominant leg, precisely 3/5 of the distance from the anterior superior iliac spine to the superior border of the patella. The scanning depth is set to the direction in which the femur can be detected. After the medial echogenic line of the rectus femoris muscle was outlined with a moving cursor on the frozen image, the thickness of the rectus femoris (RF_*thick*_) and the cross-sectional area of the rectus femoris (RF_*csa*_) were calculated. The average of the three measurements was taken as RF_*thick*_ and RF_*csa*_.

### Assessment of Sarcopenia

The definition of sarcopenia followed the guidelines from the Asian Working Group for Sarcopenia (10), as follows: low muscle mass [bioelectrical impedance (males, < 7.0 kg/m^2^; females, < 5.7 kg/m^2^)]; low muscle strength [handgrip strength (males, < 28 kg; females < 18 kg)]; and poor physical performance [5-time chair stand test (5STS), ≥ 12 s]. Muscle mass was evaluated using bioelectrical impedance analysis [BIA] (InBody770; InBody, Seoul, South Korea). Muscle strength was assessed by handgrip strength using a JAMAR^®^Plus^+^ hand dynamometer (Sammons Preston, Bolingbrook, IL, United States). For the body composition assessment, patients stood barefoot on the platform of the InBody with the soles of their feet on the electrodes. The handles of the unit were grasped with their thumb and fingers to maintain direct contact with the electrodes and remained still for ∼1 min while keeping their elbows fully extended and their shoulder joint abducted to an ∼30° angle. HGS assessment was performed using a standard technique (33, 34), with the patient in the sitting position, the elbow at 90° flexion and the wrist in a neutral position. The subjects were instructed to apply the maximum HGS 3 times with both hands, with 30 s of rest allowed between each measurement.

### Enzyme-Linked Immunosorbent Assay

Venous blood was obtained in the fasted state. Blood samples were collected into serum separator tubes, left to stand for 2 h at room temperature to clot, and then centrifuged at 3,000 rpm for 15 min at room temperature. The supernatant liquid was obtained and kept in a –80°C refrigerator. GDF15 was assessed by high-sensitivity enzyme-linked immunosorbent assay (ELISA) kits (DGD150; R&D, Minneapolis, MN, United States) following the manufacturer’s instructions.

### Statistical Analyses

Statistical analyses were performed using SPSS software (SPSS Statistics for Windows, Version 13.0. Chicago: SPSS Inc.). Continuous variables are expressed as the mean values and standard deviation. Pearson’s correlation coefficient was calculated to examine the association between continuous variables. The strengths of the correlations were weak (0–0.25), moderate (> 0.25–0.50), strong (> 0.50–0.75), and very strong (> 0.75) (35). Differences between two groups were assessed using Student’s *t*-test (normally distributed data) or the Mann–Whitney test (non-normal distribution). *p*-values < 0.05 were considered statistically significant. Receiver operating characteristic (ROC) curve analysis and the area under the curve (AUC) were used to determine the ability of serum GDF15 to predict sarcopenia. R software (version 3.4.0) and the R package “rms” were used to establish the nomogram model. ROC analysis and calibration plots were used to assess the accuracy of the predictive ability of the nomogram.

## Results

### Baseline Characteristics

Patients (*n* = 235) with stable COPD were recruited in this study, and their baseline characteristics are given in Table 1. Overall, the mean age of all patients with COPD was 64.4 ± 10.7 years, and 68% (160/235) of patients were male. The range of FEV1% of our patients was 15–108.4%, suggesting different severities of the included patients.

**TABLE 1:** Baseline characteristics of subjects.

Variable	Total (*n* = 235)	With sarcopenia (*n* = 83)	Without sarcopenia (*n* = 152)	*p*-value
Demographics				
Age, years	64.4 ± 10.7	69.2 ± 7.2	62.5 ± 13.5	0.002
Sex, male/female (*n*)	160/75	53/30	107/45	0.304
Pulmonary function				
FEV_1_, % predicted	60.5 ± 21.6	55.1 ± 22.4	64.2 ± 20.2	0.023
FVC, % predicted	82.1 ± 22.3	75.4 ± 25.1	85.6 ± 19.4	0.035
FEV_1_/FVC, %	54.2 ± 10.5	52.8 ± 11.1	57.7 ± 8.5	0.017
GOLD stage				0.006
1 + 2	156	43	113	
3 + 4	89	40	49	
Physical function				
6MWD, *m*	371.4 ± 74.4	336.0 ± 75.5	391.9 ± 65.9	<0.001
5STS, *s*	8.2 ± 3.0	9.8 ± 3.7	7.1 ± 1.9	0.001
Body composition				
BMI, kg/m^2^	23.8 ± 3.8	22.4 ± 3.5	24.8 ± 3.8	0.003
FFMI (kg/m^2^)	16.8 ± 2.3	15.6 ± 2.4	17.5 ± 2.1	0.001
FFMI (male, kg/m^2^)	17.5 ± 2.2	16.0 ± 2.3	18.2 ± 1.7	<0.001
FFMI (female, kg/m^2^)	15.0 ± 1.9	13.4 ± 2.9	15.6 ± 1.2	0.03
SMMI (kg/m^2^)	6.3 ± 1.7	5.8 ± 0.9	6.8 ± 1.1	<0.001
SMMI (male, kg/m^2^)	6.8 ± 1.0	6.2 ± 0.7	7.1 ± 1.0	<0.001
SMMI (female, kg/m^2^)	5.4 ± 0.9	5.1 ± 0.9	5.7 ± 0.7	0.024
HGS (kg)	28.1 ± 6.9	23.9 ± 7.2	30.5 ± 6.9	<0.001
HGS (male, kg)	28.9 ± 7.4	24.9 ± 6.0	30.8 ± 7.2	<0.001
HGS (female, kg)	26.9 ± 9.2	22.7 ± 8.4	30.2 ± 8.6	0.016

*FEV1, forced expiratory volume in the first second; FEV1% pred, FEV percentage predicted; FVC, forced vital capacity; FVC% pred, FVC percentage predicted; GOLD, Global Initiative for Chronic Obstructive Lung Disease; 6MWD, 6-min walk distance; 5STS, five-time chair stand test; BMI, body mass index; FFMI, fat-free mass index; SMMI, skeletal muscle mass index; HGS, handgrip strength.*

Next, we analyzed the difference between patients with sarcopenia and patients without sarcopenia. As shown in Table 1, patients with sarcopenia were significantly older. Pulmonary function (FEV_1_%predicted, FVC% predicted, and FEV_1_/FVC) was significantly decreased in sarcopenic patients with COPD. Patients with sarcopenia had significantly advanced GOLD stages. In addition, patients with sarcopenia showed poor physical function (6MWD and 5STS). In terms of body composition, BMI, FFMI, SMMI, and HGS were markedly decreased in sarcopenic patients.

A total of 235 patients were assigned to the development set (117 cases) and validation set (118 cases), and the clinical features of patients in these two sets were not significantly different (Table 2).

**TABLE 2:** Patient characteristics of the development set and validation set.

Variable	Development set (*n* = 117)	Validation set (*n* = 118)	*p*-value
Demographics			
Age, years	64.9 ± 12.0	63.9 ± 9.2	0.110
Sex, m/f (*n*)	83/34	77/41	0.349
Pulmonary function			
FEV_1_,%predicted	59.8 ± 21.0	56.2 ± 20.7	0.368
FVC,% predicted	82.5 ± 23.7	84.5 ± 20.0	0.700
FEV_1_/FVC, %	55.2 ± 9.9	53.1 ± 11.1	0.232
GOLD stage			0.927
1 + 2	78	78	
3 + 4	39	40	
Physical function			
6MWD, *m*	369.0 ± 74.2	359.1 ± 78.1	0.370
5STS, *s*	7.8 ± 3.3	7.4 ± 2.3	0.624
Body composition			
BMI, kg/m^2^	23.9 ± 3.9	23.6 ± 3.8	0.532
FFMI (kg/m^2^)	16.8 ± 2.4	16.7 ± 2.3	0.750
FFMI (male, kg/m^2^)	17.5 ± 2.2	17.4 ± 2.0	0.956
FFMI (female, kg/m^2^)	15.0 ± 2.0	15.2 ± 2.4	0.798
SMMI (kg/m^2^)	6.4 ± 1.1	5.9 ± 1.4	0.143
SMMI (male, kg/m^2^)	6.8 ± 1.0	6.5 ± 1.1	0.470
SMMI (female, kg/m^2^)	5.4 ± 0.9	5.1 ± 1.4	0.436
HGS (kg)	28.2 ± 8.7	28.0 ± 7.6	0.787
HGS (male, kg)	29.4 ± 8.0	28.4 ± 6.9	0.699
HGS (female, kg)	26.1 ± 9.9	26.6 ± 9.8	0.817

*FEV_1_, forced expiratory volume in the first second; FEV_1_% pred, FEV percentage predicted; FVC, forced vital capacity; FVC% pred, FVC percentage predicted; GOLD, Global Initiative for Chronic Obstructive Lung Disease; 6MWD, 6-min walk distance; 5STS, five-time chair stand test; BMI, body mass index; FFMI, fat-free mass index; SMMI, skeletal muscle mass index; HGS, handgrip strength.*

### Correlation Between Serum GDF15 Levels and Clinical Features

**FIGURE 1:**
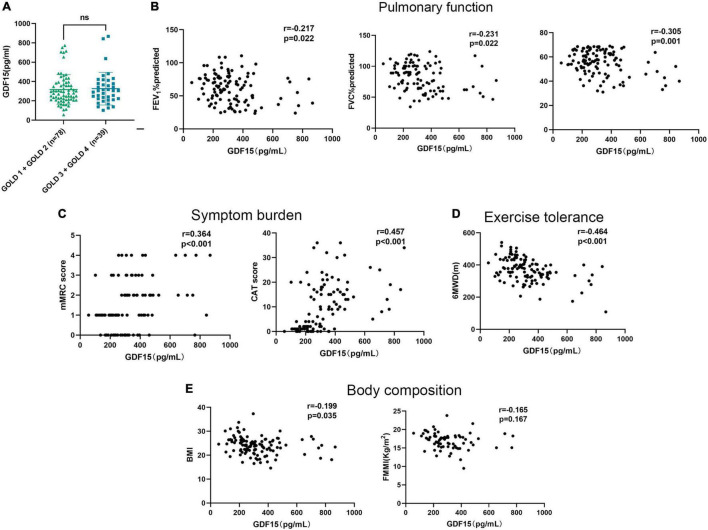
The relationships of serum GDF15 levels with the clinical features of patients with COPD. **(A)** Difference in serum GDF15 level in patients with GOLD A and GOLD B and in patients with GOLD C and GOLD D; **(B)** the relationship between serum GDF15 level and FEV_1_%predicted, FVC % predicted, FEV_1_/FVC (*n* = 117); **(C)** the relationship between serum GDF15 level and mMRC score, CAT score (*n* = 117); **(D)** the relationship between serum GDF15 level and 6MWD; **(E)** the relationship between serum GDF15 level and BMI, FFMI (*n* = 117). GOLD, global initiative for chronic obstructive lung disease; FEV_1_%predicted, forced expiratory volume in the first second percentage predicted; FVC% predicted, forced vital capacity percentage predicted; mMRC, the modified British Medical Research Council; CAT, COPD assessment test; 6MWD, 6-min walking distance; BMI, body mass index; FFMI, fat-free mass index.

Serum GDF15 expression levels were not significantly different between male (323.9 ± 162.4 pg/mL) and female (287.7 ± 111.6 pg/mL) patients (*p* = 0.633). First, we found that serum GDF15 expression levels were not associated with GOLD stage in patients with COPD (Figure 1A). Furthermore, we analyzed its correlation with pulmonary function, and as shown in Figure 1B, serum GDF15 levels were significantly negatively correlated with FEV_1_% predicted (*r* = –0.217, *p* = 0.022), FVC% predicted (*r* = –0.231, *p* = 0.022), and FEV_1_/FVC (*r* = –0.305, *p* = 0.001). In addition, we found that GDF15 levels were associated with worsening clinical symptoms and health status (Figure 1C). This was confirmed by the significant negative correlation between serum GDF15 levels, mMRC score (*r* = 0.364, *p* < 0.001), and CAT score (*r* = 0.457, *p* < 0.001). As shown in Figure 1D, we found that a higher serum GDF15 level was associated with a lower 6MWD (*r* = –0.464, *p* < 0.001). Finally, we analyzed the relationship between serum GDF15 levels and body composition, and as shown in Figure 1E, serum GDF15 levels were negatively associated with BMI (*r* = –0.199, *p* = 0.035) and not related to FFMI (*r* = –0.165, *p* = 0.167).

### Relationships Between Serum GDF15 Levels and Skeletal Muscle Function in Patients With Chronic Obstructive Pulmonary Disease

**FIGURE 2:**
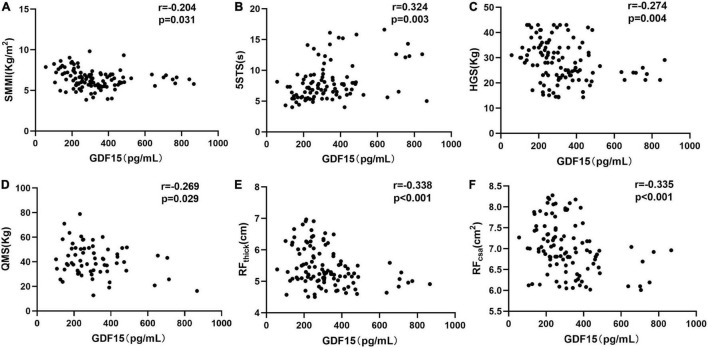
The relationships of serum GDF15 levels with skeletal muscle function in patients with COPD. **(A)** The relationship between serum GDF15 levels and SMMI (*n* = 117; **(B)** the relationship between serum GDF15 levels and 5STS (*n* = 117); **(C)** the relationship between serum GDF15 levels and HGS (*n* = 117); **(D)** the relationship between serum GDF15 levels and QMS (*n* = 111); **(E,F)** the relationship between serum GDF15 levels and HGS (*n* = 117); **(D)** the relationship between serum GDF15 levels and RF_thick_, RF_csa_ (*n* = 114). SMMI, Skeletal muscle mass index; 5STS, Five-time chair stand test; HGS, Handgrip strength; QMS, Quadriceps muscle strength; RF_thick_, The thickness of the rectus femoris, RF_thick_ **(E)**, RF_csa_, The cross-sectional area of rectus femoris.

Next, we aimed to determine the relationship between serum GDF15 levels and skeletal muscle function, and as shown in Figure 2A, serum GDF15 levels were negatively associated with skeletal muscle mass (*r* = –0.204, *p* = 0.031). The 5STS test is used to assess physical strength during longer periods, suggesting poorer physical performance (13). In Figure 2B, serum GDF15 levels were positively correlated with 5STS (*r* = 0.324, *p* = 0.003). Furthermore, we analyzed the relationship between serum GDF15 levels and muscle strength. HGS and quadriceps muscle strength (QMS) are common indicators of muscle strength. In Figures 2C,D, the serum GDF15 levels were negatively correlated with HGS (*r* = –0.274, *p* = 0.004) and QMS (*r* = –0.269, *p* = 0.029). Finally, we applied ultrasound to measure the thickness and area of the rectus femoris muscle in patients with COPD, and as shown in Figures 2E,F, serum GDF15 levels were negatively correlated with RFthick (*r* = –0.338, *p* < 0.001) and RFcsa (*r* = –0.335, *p* < 0.001). Overall, these results indicated that GDF15 is a potential important biomarker in patients with COPD reflecting skeletal muscle function.

GDF15 has been proposed as a novel biomarker of biological aging in humans. Therefore, univariate analysis and multivariate analysis were used to explore the association between age, GDF15, and sarcopenia in patients with COPD. The results of univariate analysis showed that old age (odds ratio [OR]: 1.084, 95% CI: 1.028–1.144, *p* = 0.003) and elevated serum GDF15 levels (OR: 5.396, 95% CI: 2.313–12.588, *p* < 0.001) were associated with sarcopenia in patients with COPD. Multivariate analysis showed that old age (OR: 1.125, 95% CI: 1.036–1.222, *p* = 0.005) and elevated serum GDF15 levels (OR: 5.069, 95% CI: 3.143–8.980, *p* < 0.001) were independent risk factors. These results suggest that the association of serum GDF15 levels with sarcopenia was independent of age.

### The Clinical Value of Serum GDF15 Levels for the Prediction of Sarcopenia in Patients With Chronic Obstructive Pulmonary Disease

**FIGURE 3:**
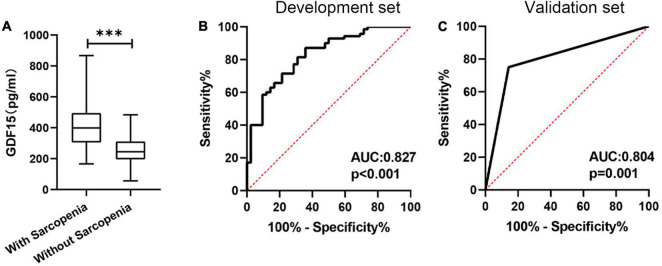
The predictive value of the serum GDF15 level. **(A)** The serum GDF15 level differed between patients with sarcopenia and patients without sarcopenia; **(B)** receiver operating characteristic curve analysis of serum GDF15 level for the prediction of sarcopenia in the development set; **(C)** receiver operating characteristic curve analysis of serum GDF15 level for the prediction of sarcopenia in the validation set. ^***^p < 0.001.

Next, we analyzed serum GDF15 levels to assess its diagnostic significance for the prediction of sarcopenia in patients with COPD. The definition of sarcopenia followed the guidelines from the Asian Working Group for Sarcopenia, as follows: low muscle mass (bioelectrical impedance [males, < 7.0 kg/m2; females, < 5.7 kg/m2]); low muscle strength (handgrip strength [males, < 28 kg; females < 18 kg]); and poor physical performance (5STS, ≥ 12 s). First, we found that GDF15 in patients with sarcopenia was significantly higher than that in controls (438.3 ± 180.9 pg/mL vs. 269.1 ± 104.3 pg/mL, *p* < 0.001) (Figure 3A). The ROC curves derived from the development set demonstrating the ability of serum GDF15 to predict sarcopenia are shown in Figure 3B. The sensitivity and specificity for predicting sarcopenia based on the serum levels of GDF15 were 65.12 and 87.84%, respectively (the cutoff point was 357.5 pg/mL, and the AUC value was 0.827). Furthermore, we verified the clinical predictive value and cutoff points of GDF15 in the validation set. As shown in Figure 3C, the AUC value for predicting sarcopenia with GDF15 was 0.801 (*p* < 0.001).

### Construction of a Nomogram to Predict Sarcopenia

**FIGURE 4:**
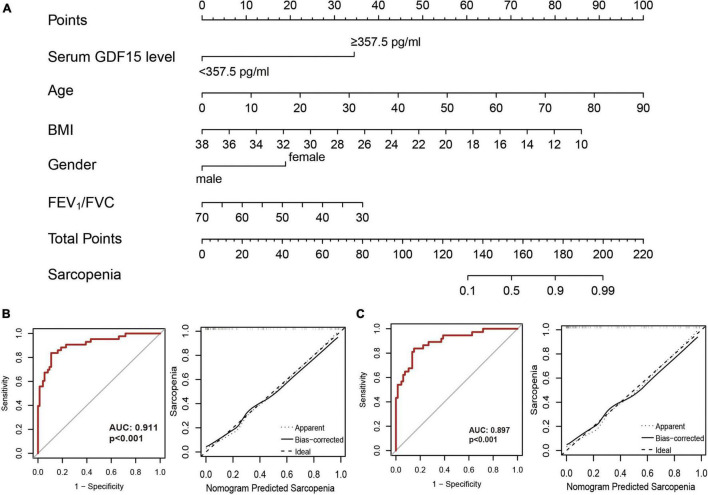
Construction of nomogram models. **(A)** A nomogram combining serum GDF15 level and clinical features was constructed based on the development set; **(B)** receiver operating characteristic curve analysis (left) and decision curve analysis (right) in the development set; **(C)** receiver operating characteristic curve analysis (left) and decision curve analysis (right) in the validation cohort.

To further improve the predictive efficacy and clinical applicability, we constructed a nomogram, as shown in Figure 4A, serum GDF15 levels, age, BMI, gender, and FEV_1_/FVC were used for the construction of the nomogram model based on the development set. The calibration curve showed good agreement among the estimates obtained with the nomogram and actual observations, and the AUC value of the nomogram model was 0.911 based on the results of the ROC analysis (Figure 4B). The calibration curve and ROC analysis (AUC = 0.897) based on the validation cohort also confirmed the predictive ability of the nomogram model (Figure 4C).

## Discussion

To our knowledge, this is the first study to assess the clinical value of serum GDF15 levels in patients with COPD for the prediction of sarcopenia. We found for the first time that serum GDF15 levels represent a simple and efficient indicator of sarcopenia in COPD patients. In addition, we determined the cutoff value for serum GDF15 levels and confirmed this within a validation set. Thus, serum GDF15 levels may accurately predict sarcopenia in COPD patients. The strengths of this prospective observational study are that we showed that the serum GDF15 level is simple and useful as a screening test for sarcopenia in patients with COPD and nomogram model based on GDF15 level and clinical features could further improves the predictive ability.

GDF15 is a member of the transforming growth factor β superfamily (15) and is mainly involved in cell growth, differentiation, development and tissue repair (18, 36). Under physiological conditions, GDF15 expression is low in all tissues except the placenta (37). GDF15 expression is upregulated under pathological conditions such as inflammation and various stimulatory factors (TGF-β, IL-1β, TNF-α, etc.) (38). GDF15 inhibits lipopolysaccharide-induced TNF-α production, suggesting that it could inhibit the activation of macrophages (39). In addition, the expression of hepatic GDF15 in NAFLD directly correlates with IL-1β content and the severity of steatosis (40). Therefore, GDF15 could be used as an inflammatory marker or stress response protein. Cigarette smoking (CS) extracts could induce GDF15 expression in human small airway epithelial cells (41). In addition, deletion of GDF15 in mice diminished CS-associated lung inflammation (42). GDF15 also plays an important role in airway mucosal immunity, as it promotes mucin production by activating phosphatidylinositol 3-kinase (PI3K) in ciliated epithelial cells (41, 43). GDF15 induction by CS also promotes cellular senescence through the activin receptor-like kinase 1/Smad1 pathway and increases the expression of cellular senescence markers, including p21, p16, and high mobility group box 1 (HMGB1), in airway epithelial cells (44). Overall, GDF15 plays an important pathophysiological role in cancer, cardiovascular disease, COPD, etc.

In this study, we also found that serum GDF15 levels were weakly associated with pulmonary function, moderately associated with clinical symptoms, and weakly associated with body composition in patients with COPD, and our results are consistent with previous reports (25, 45). Inadequate exercise tolerance is an important manifestation in patients with COPD (46, 47), and 6MWD < 350 m is an independent risk factor for patients with COPD (48, 49). Our results found that serum GDF15 levels were moderately associated with exercise tolerance. Decreased physical performance is an important feature of patients with COPD and is caused by skeletal muscle dysfunction (50, 51). Our results suggest that serum GDF15 levels were moderately associated with physical performance. Collectively, these findings suggest that serum GDF15 levels may serve as clinically feasible biomarkers for the prediction of sarcopenia in patients with COPD. The strength of our study was that we collated a more comprehensive set of patient characteristics so that we were able to fully assess the potential clinical value of GDF15 in patients with COPD at the same baseline level. In addition, a larger sample size was included in the present study than in previous studies. Our results further demonstrated the potential clinical value of GDF15 as a biomarker in patients with COPD.

A previous study (45) from patients with COPD showed higher GDF15 in patients with cachexia, and they showed no effect of GDF15 levels on the rate of decline in FFMI. This is consistent with our results. Our results also indicated that serum GDF15 levels were not related to FFMI (*r* = –0.165, *p* = 0.167). Husebø et al. (45) found that patients were mainly in an advanced stage (half of the patients were in GOLD stage 3 or 4). Our study included more patients in early stages (2/3 of the subjects were in GOLD stage 1 or 2). Overall, these results may suggest that GDF15 is not associated with FFMI.

Recently, circulating factors have been identified as potential biomarkers for sarcopenia in patients with COPD, and several studies have indicated that antioxidant and oxidant biomarkers (52), systemic inflammation biomarkers (53), muscle damage biomarkers, and miRNAs (54) are correlated with muscle mass and strength in patients with COPD. Qaisar et al. (55) constructed a cumulative risk score based on the serum levels of six circulating biomarkers, and the risk score could divide patients into high- and low-risk groups for sarcopenia, with an AUC value of 0.793. Hirai et al. (56) found that the serum creatinine/cystatin C ratio could serve as a predictor of sarcopenia in patients with COPD. However, these biomarkers cannot currently be used in clinical practice due to insufficient sample sizes and predictive power and a lack of external verification. Our results from the development set and validation set indicated that higher serum GDF15 levels (> 357.5 pg/mL) showed a good predictive ability for sarcopenia. This study was the first to clarify the predictive value of serum GDF15 levels for sarcopenia in patients with COPD. Importantly, we determined the cutoff point of serum GDF15 levels, which will contribute to clinical application.

Our data show that serum GDF15 levels can effectively distinguish patients with COPD with sarcopenia, and we also determined the cutoff value. However, the application of GDF15 levels and cutoff points to predict sarcopenia in patients with COPD warrants caution in clinical practice. Serum GDF15 levels in patients with COPD are significantly higher than those in healthy controls. However, the GDF15 level (approx. 300 pg/mL) of patients with COPD in this study was lower than the healthy control (approximately 400–500 pg/mL) in previous studies from the Caucasian population (22, 45), Chinese population (57, 58), etc. A previous study (59) indicated a clear dose–response association between serum GDF-15 concentrations and dietary patterns. Different populations and different regions of China have significantly different dietary habits. The participants in this study were all from Northern China. Therefore, we consider that these differences in GDF15 levels may be due to population and district. Caution should be taken when extrapolating the clinical application of GDF15 to other districts and ethnicities. Overall, the cutoff value of GDF15 may be applicable to patients with COPD in northern China. Multicenter, prospective studies are needed to establish a standardized detection process and determine the cutoff value of GDF15.

GDF15 expression is very low in healthy individuals and young subjects (22, 37). The levels of GDF15 dramatically increase in chronic or acute illness conditions, in the presence of age-related diseases, as well as with aging independent of the health state; thus, GDF15 has been proposed as a novel biomarker of biological aging in humans (60, 61). Sarcopenia is an age-related disease, and with the aging of the human body, there is inevitably a gradual decline in muscle mass, quality, and strength (62). In this study, we demonstrated that the association of serum GDF15 levels with sarcopenia in patients with COPD was independent of age.

Single biomarkers lack sufficient predictive efficacy, and predictive models covering multiple biomarkers can significantly improve predictive power. Therefore, we constructed nomograms (combining patient clinical characteristics and serum GDF15 levels) and showed good predictive power in the development set and validation set. Nomograms are a statistical modeling tool that integrates the effects of various clinical factors and are widely used to assist clinicians in decision making (32). Age, BMI, and FEV_1_/FVC were included in the construction of this nomogram because previous studies (8) have shown that these clinical features were significantly associated with the risk of sarcopenia in patients with (22) COPD. We also included gender into this model because muscle wasting has a greater impact on women with COPD. The nomogram model we constructed further improves the predictive ability of identifying patients with sarcopenia and is more visual. The involvement of more proven and accessible biomarkers in the nomogram model could further improve the predictive power. However, due to the limitation of the volume of clinical samples, we were unable to verify more biomarkers (systemic inflammation biomarkers, serum creatinine/cystatin C ratio, etc.) into the nomogram model. Therefore, the inability to incorporate more proven and accessible biomarkers is one of the limitations of our study.

The present study also has several limitations. First, the inability to obtain potential clues for GDF15 through database and bioinformatics screening is one of the main limitations of this study. Recent advances in omics techniques, including genomics, transcriptomics, proteomics, and metabolomics, offer new opportunities to identify novel biomarkers. In future research, we will try to use multiomics data to explore new biomarkers. Second, BIA used to measure muscle mass is widely used for the diagnosis of sarcopenia but is not a good surrogate for skeletal muscle mass. Furthermore, there was a lack of healthy controls in this study, and several studies (45, 63) have shown that GDF15 levels are increased in patients with COPD when compared to healthy subjects. In addition, this study was limited to clinically stable patients with COPD. The lack of patients with acute exacerbations of COPD is one of the main limitations of this study. Previous studies (63, 64) indicated that GDF-15 is elevated by exacerbation of COPD and could serve as a novel blood biomarker of acute exacerbation of COPD that is more sensitive than that of CRP. Muscle loss accelerates in patients during acute exacerbations of COPD (65). Therefore, more evidence is still needed to confirm whether serum GDF15 levels can be applied to identify sarcopenia in patients with acute exacerbations of COPD. Finally, whether these findings can be applied to patients undergoing pulmonary rehabilitation remains to be determined.

## Conclusion

In conclusion, serum GDF15 levels can be used to accurately and easily evaluate sarcopenia in patients with COPD. The nomogram models based on serum GDF15 level, age, and BMI further improved the predictive ability of identifying patients with sarcopenia.

## Data Availability Statement

The original contributions presented in this study are included in the article/Supplementary Material, further inquiries can be directed to the corresponding author/s.

## Ethics Statement

The study was approved by the Research Ethics Committees of the First Hospital of China Medical University (No. 2018-144-2) and was approved by Ethics Committees at the First Hospital of Dalian Medical University. All patients signed informed consent forms to allow analyses to be performed on their tissue samples.

## Author Contributions

GH: conceptualization. MD: data curation. YB and QZ: methodology. XZ: project administration. MD and GH: writing—original draft, review and editing. All authors contributed to the article and approved the submitted version.

## Conflict of Interest

The authors declare that the research was conducted in the absence of any commercial or financial relationships that could be construed as a potential conflict of interest.

## Publisher’s Note

All claims expressed in this article are solely those of the authors and do not necessarily represent those of their affiliated organizations, or those of the publisher, the editors and the reviewers. Any product that may be evaluated in this article, or claim that may be made by its manufacturer, is not guaranteed or endorsed by the publisher.

## Supplementary Material

Supplementary Figure 1 Flow diagram.
