# Supplementary figures and images for: Growth Differentiation Factor-15 as a Biomarker for Sarcopenia in Patients With Chronic Obstructive Pulmonary Disease
Source: Front Nutr. 2022 Jun 30;9:897097. doi: 10.3389/fnut.2022.897097 (PMC9282868; doi:10.3389/fnut.2022.897097)

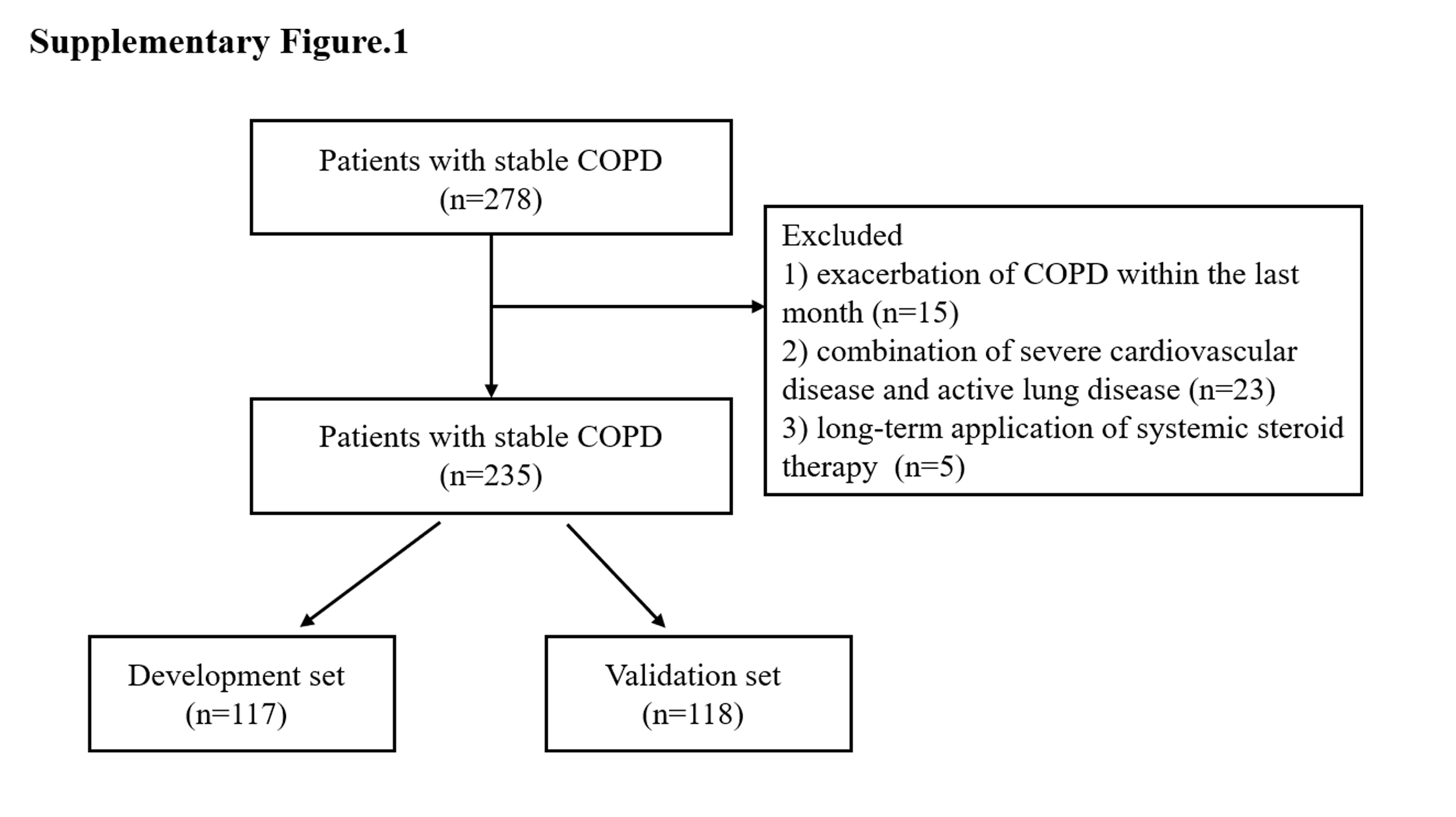

Supplement: Supplementary Figure 1 — Flow diagram. [file Image_1.JPEG]
